# OPTimal IMAging strategy in patients suspected of non-traumatic pulmonary disease at the emergency department: chest X-ray or ultra-low-dose CT (OPTIMACT)—a randomised controlled trial chest X-ray or ultra-low-dose CT at the ED: design and rationale

**DOI:** 10.1186/s41512-018-0038-1

**Published:** 2018-08-08

**Authors:** Inge A. H. van den Berk, Maadrika M. N. P. Kanglie, Tjitske S. R. van Engelen, Shandra Bipat, Marcel G. W. Dijkgraaf, Patrick M. M. Bossuyt, Wouter de Monyé, Jan M. Prins, Jaap Stoker

**Affiliations:** 1 Radiology and Nuclear Medicine, Amsterdam Gastroenterology and Metabolism, Cancer Center Amsterdam, Amsterdam UMC, location AMC, University of Amsterdam, Amsterdam, Netherlands; 2 Internal Medicine, Infection and Immunity: Infectious Diseases, Amsterdam UMC, location AMC, University of Amsterdam, Amsterdam, Netherlands; 3 Clinical Epidemiology, Biostatics and Bioinformatics, Amsterdam Public Health: Methodology / Personalized Medicine, Amsterdam UMC, location AMC, University of Amsterdam, Amsterdam, Netherlands; 4 Radiology, Spaarne Gasthuis, Haarlem, Netherlands

**Keywords:** Ultra-low-dose chest CT, ULD chest CT, Microdose chest CT, Chest X-ray, Non-traumatic pulmonary disease, Non-traumatic chest disease, Pulmonary disease, Emergency department

## Abstract

**Background:**

Chest X-ray has been the standard imaging method for patients suspected of non-traumatic pulmonary disease at the emergency department (ED) for years. Recently, ultra-low-dose chest computed tomography (ULD chest CT) has been introduced, which provides substantially more detailed information on pulmonary conditions that may cause pulmonary disease, with a dose in the order of chest X-ray (0.1 vs. 0.05 mSv). The OPTimal IMAging strategy in patients suspected of non-traumatic pulmonary disease at the emergency department: chest X-ray or CT (OPTIMACT) study is a randomized trial designed to evaluate the effectiveness of replacing chest X-ray for ULD chest CT in the diagnostic work-up of patients suspected of non-traumatic pulmonary disease at the ED.

**Methods:**

Two thousand four hundred patients presenting at the ED with pulmonary complaints and suspected of non-traumatic pulmonary disease will be enrolled in this multicenter, pragmatic, randomized trial. During randomly assigned periods of one calendar month, either conventional chest X-ray or ULD chest CT scan will be used as the imaging strategy. Randomization will rely on computer-generated blocks of 2 months to control for seasonal effects. Chest X-ray and ULD chest CT will be performed in a standardized way, after obtaining the clinical history and performing physical examination and initial laboratory tests. The primary outcome measure is functional health at 28 days. Secondary outcome measures are mental health, length of hospital stay, mortality within 28 days, quality-adjusted life years (QALYs) during the first 28 days, correct diagnoses at ED discharge as compared to the final post hoc diagnosis, and number of patients in follow-up because of incidental findings on chest X-ray or ULD chest CT. In an economic evaluation, we will estimate total health care costs during the first 28 days.

**Discussion:**

This pragmatic trial will clarify the effects of replacing chest X-ray by ULD chest CT in daily practice, in terms of patient-related health outcomes and costs, in the diagnostic work-up of patients suspected of non-traumatic pulmonary disease at the ED.

**Trial registration:**

The OPTIMACT trial is registered in the Netherlands National Trial Register under number NTR6163. The date of registration is December 6, 2016.

**Electronic supplementary material:**

The online version of this article (10.1186/s41512-018-0038-1) contains supplementary material, which is available to authorized users.

## Background

Dyspnea is a common complaint among patients attending the emergency department (ED). In 2011, dyspnea was the chief complaint in 3.7 million visits, or 2.7%, of more than 136 million visits to the US EDs [1]. Dyspnea-related chief complaints (cough, chest discomfort) contributed to 8.2% of ED visits [1]. These patients are suspected of non-traumatic pulmonary disease, and chest X-ray is a standard diagnostic procedure. Chest X-ray helps to elucidate important causes for pulmonary complaints, such as pneumonia, congestion, and pneumothorax, at a very low ionizing radiation dose (0.05 mSv).

Being a two-dimensional projection technique, chest X-ray has obvious limitations, e.g., missed pneumonia by overlying heart or ribs obscuring lung pathology [2–9]. As a cross-sectional three-dimensional imaging technique, computed tomography (CT) better highlights chest anatomy and pathology [10–12]. Because of the much higher radiation dose (5 mSv), standard chest CT is not suitable for routine imaging in dyspneic patients [13, 14]. The radiation dose of CT is a serious concern, as it is a major factor contributing to the annual ionizing radiation exposure of the population. In the USA, the average radiation dose per person is 6.2 mSv, of which 48% is caused by medical imaging, mainly CT [15]. For this reason, current guidelines do not recommend CT scanning as a standard imaging technique in patients suspected of non-traumatic pulmonary disease [16, 17].

Recently, iterative reconstruction has been introduced, allowing the introduction of CT scanners with intrinsically lower radiation exposure for any application, compared to current machines. These scanners are becoming the new standard in CT technology [18].

The new scanners are also capable of acquiring ultra-low-dose chest CT (ULD chest CT) [19–21]. Compared to chest X-ray, ULD chest CT provides substantially more detailed information on pulmonary conditions that may cause dyspnea, with a dose in the order of chest X-ray (0.1 vs. 0.05 mSv) and even lower doses seem in reach. Hence, compared to standard chest X-ray, this ULD chest CT may lead to more timely diagnoses, more timely treatment, and improved patient management. The image quality of ULD chest CT is less than the standard chest CT, but ULD chest CT gives a high level of diagnostic confidence in patients suspected of pulmonary disease at the ED [22].

Until now research on ULD chest CT was mainly focused on lung cancer screening and cardiovascular imaging [23–26]. There have been several studies investigating the use of ULD chest CT in patients with neutropenic fever [27]. In community-acquired pneumonia (CAP), preliminary data also suggested an increased sensitivity of standard CT scanning [2, 4, 7, 11, 13]; 33% of infiltrates identified by CT scan were not found on chest X-ray [10]. A recent study in 319 patients convincingly showed that early standard CT scan findings markedly improved diagnostic accuracy compared to chest X-ray. Chest radiograph revealed a parenchymal infiltrate in 188 patients. CT scan revealed a parenchymal infiltrate in 40 (33%) patients without an infiltrate on the chest radiograph and excluded CAP in 56 (29.8%) of the 188 with parenchymal infiltrate on the radiograph. In 14% of patients, administration of antibiotics was stopped, whereas in 46%, treatment with antibiotics was started based on these results. It was unclear whether the improved diagnostics and the changes in management resulted in better outcome [10]. The role of CT in diagnoses other than CAP in patients suspected of non-traumatic pulmonary disease at the ED is unclear.

On the other hand, incidental findings on CT may lead to additional examinations, which can increase costs and uncertainty, without contributing to patient outcome in most patients. One study on incidental findings on chest CT performed between 2006 and 2012 in the general population reported that nodules were found in 24 to 31% of all chest CT scans [28]. In a lung cancer screening trial in (previous) smokers, 1729 of 2994 participants (58%) had at least one non-calcified nodule. The number of nodules per patient varied from 1 to 20, resulting in a total of 4026 nodules. Twenty percent of these nodules were identified as peri-fissural nodules, i.e., intrapulmonary lymph nodes, which are completely benign findings without an indication for follow-up. This implies that about four out of five of those with at least one non-calcified nodule required follow-up in that trial [29]. However, these populations have an a priori higher risk of acquiring a pulmonary malignancy because of smoking habits and age distribution compared to the more clinically diverse target population presenting at the ED with non-traumatic pulmonary complaints.

In summary, preliminary data suggest an increased sensitivity of chest CT scanning at the ED compared to chest X-ray, which is possibly accompanied by more incidental findings. To what extent this translates into an improvement in patient outcomes is yet unknown.

The purpose of this study, the OPTIMACT trial (OPTimal IMAging strategy in patients suspected of non-traumatic pulmonary disease at the emergency department: chest X-ray or ultra-low-dose CT), is to evaluate the effectiveness of replacing chest X-ray for ULD chest CT on patient outcomes in the diagnostic work-up of patients suspected of non-traumatic pulmonary disease at the ED. The hypothesis is that more accurate imaging will allow a timelier diagnosis and timelier treatment and will improve patient management, resulting in increased efficiency and lower costs, with at least comparable functional health.

## Methods/design

During the design of the protocol, the SPIRIT recommendations were used [30], (Additional file 1: Appendix 1).

### Study objectives

The primary goal is to evaluate the effects, in terms of patient-related health outcomes and cost-effectiveness, of replacing chest X-ray by ULD chest CT in the diagnostic work-up of patients suspected of non-traumatic pulmonary disease at the ED.

The secondary goal is to evaluate whether the replacement of chest X-ray by ULD chest CT leads to more accurate diagnoses at ED discharge and more timely treatment.

For patients with clinically suspected CAP, additional objectives are to evaluate the diagnostic accuracy and clinical impact of performing ULD chest CT as compared to conventional chest X-ray and the accuracy of ULD chest CT versus conventional X-ray and to predict the etiology of pneumonia.

### Study design

OPTIMACT is a multicenter, pragmatic, non-inferiority randomized controlled trial (RCT) comparing ULD chest CT to chest X-ray in consecutive patients suspected of non-traumatic pulmonary disease presenting at the ED.

During randomly assigned periods of one calendar month, either conventional chest X-ray or ULD chest CT scan will be used as the imaging strategy. The strategies will be randomized using computer-generated blocks of 2 months to control for seasonal influence on pulmonary disease caused by the influenza period. As for including physicians from various departments (i.e., emergency medicine, cardiology, pulmonology, and internal medicine) have rotating residents at the EDs, a large number of medical doctors need to be educated and informed continuously about the study. A monthly randomization also from this perspective is the most feasible option in this situation.

Data on the effects of diagnostic imaging on patient outcomes are commonly regarded as higher-level evidence, compared to diagnostic accuracy [31]. A more accurate diagnosis should result in more timely treatment, and one can anticipate that this should result in patient outcomes that are at least equivalent, if not better, while reducing health care costs. Our goal is therefore to investigate the effects on functional health and healthcare costs of using ULD chest CT rather than chest X-ray in the early work-up of patients suspected of non-traumatic pulmonary disease at the ED.

### Setting

Patients are enrolled in two participating Dutch hospitals, one university hospital (Amsterdam University Medical Centers (Amsterdam UMC), location Academic Medical Center (AMC), Amsterdam) and one large teaching hospital (Spaarne Gasthuis (SG), enrolling at both locations (Haarlem and Hoofddorp)).

### Study group

The study group comprises consecutive adult patients who are self-referrals or are referred by a general practitioner or their treating physician at the hospital to the ED with suspected non-traumatic pulmonary disease: complaints of dyspnea, fever, chest pain, or cough. As the disease spectrum of pulmonary diseases at the ED varies during the day, 24/7 inclusion is mandatory.

### Eligibility criteria

#### Inclusion criteria

The inclusion criterion is patients ≥ 18 years presenting at the ED with a suspicion of non-traumatic pulmonary disease, i.e., presenting with dyspnea, fever, chest pain, or cough, in whom chest X-ray is required for work-up by the attending physician.

#### Exclusion criteria

The following are the exclusion criteria:

- Patients who are not able to undergo a chest X-ray or ULD chest CT (e.g., not able to lay in supine position)
- Incapacitated patients
- Pregnancy
- Life expectancy less than 1 month
- Patients with anticipated barriers to complete follow-up data collection
- Earlier participation in this RCT
- No informed consent for participation in study.

### Eligibility criteria for inclusion in the CAP sub-study

#### Inclusion criteria

For suspected CAP, the inclusion criteria are based on the following:

- New onset of systemic infection, i.e., at least one of the following: chills or temperature > 38 or < 36 °C
- Clinical signs and symptoms of an acute lower respiratory tract infection, i.e., at least one of the following: cough, sputum production, dyspnea, chest pain, or abnormal breathing sounds at auscultation suggestive of pneumonia.

#### Exclusion criteria

The following are the exclusion criteria

- Any other active infection requiring antibiotic treatment
- Admission to the intensive care unit (ICU)
- Pneumonia developing within 14 days of hospital discharge.

### Ethics and informed consent

The Medical Ethics Committee (MEC) of the AMC approved our final study protocol. The MEC of the other hospital (SG) approved their participation in this trial. Written informed consent will be obtained by the attending physician at the ED from eligible patients for participation in the study and using individual patient data for study purposes (Additional file 2: Appendix 2).

If the patient is incapacitated, for example, an altered mental status due to illness, the attending physician will obtain oral informed consent by reading a short patient information folder, which will be signed by a witness (e.g., a nurse not involved in the study team) (Additional file 3: Appendix 3). Consent will be registered in the electronic patient record. Once the condition of the patient has improved, the regular patient information folder can be read, and written informed consent will be obtained from the patient.

Patients who do not want to participate in the study will have a regular work-up at the ED with a chest X-ray and will get the regular standard of care. It is not possible to order a ULD chest CT as the first line imaging in this situation.

Participants can leave the study at any time for any reason if they wish to do so without any consequences. The investigator can decide to withdraw a subject from the study for urgent medical reasons. Patients withdrawing permission to use their data will not be replaced. Patients withdrawn from the RCT will receive standard medical care. Maximal efforts will be made to obtain information required for our primary endpoints from patients who will drop out of the RCT.

In the study protocol, a date and version identifier is used for the amendments made during the study to give insight in changes made and to prevent confusion between versions.

The investigational product, ULD chest CT, is a non-invasive imaging method part of standard care and under intensive quality control as part of its clinical applications. Safety reporting is therefore limited to events possibly related to the study procedure (chest X-ray vs. ULD chest CT) and only for the period the patient is admitted to the ED. There is no data safety monitory board (DSMB) because there are no added risks. Because of the large number of patients that will be included in the study, there is data monitoring of the OPTIMACT study to ensure adherence to the procedures defined in the study protocol. The monitoring is performed by the Clinical Research Unit (CRU) of the Amsterdam UMC, location AMC, that is independent from the sponsor and has no competing interest.

### Study procedures

**Fig. 1:**
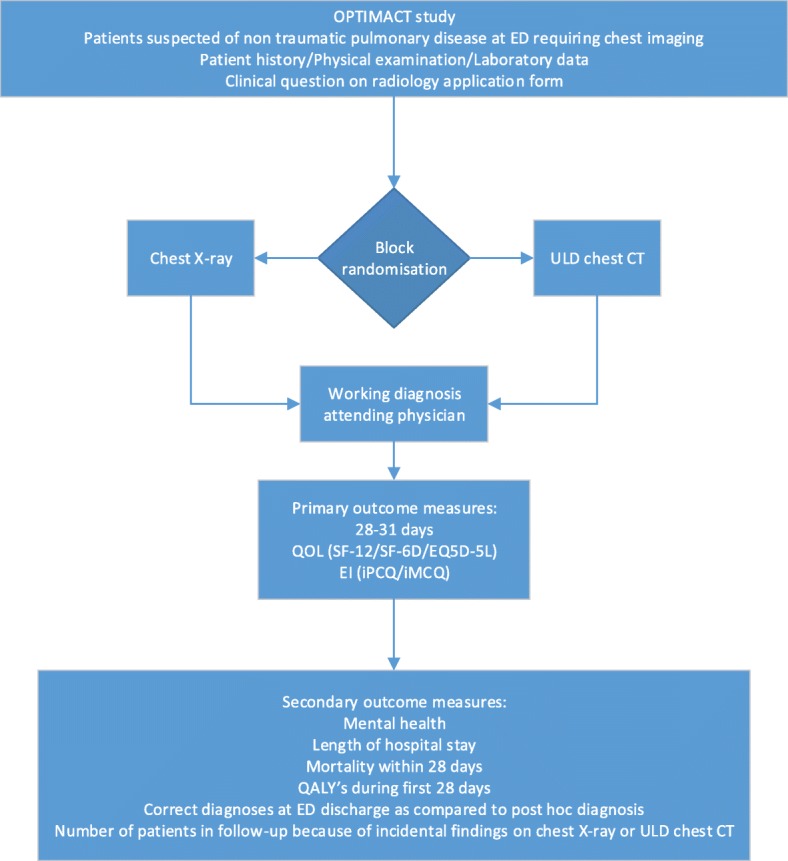
OPTIMACT study flow chart

Initial examination will consist of a standardized clinical history and physical examination, including mental state. Clinical history and physical examination are registered in a standardized format in the electronic patient record (EPIC). A predefined laboratory set is ordered. The attending physician will evaluate this information and formulate a working diagnosis and probability on the radiology application form (Fig. 1).

The imaging method that is randomized that month, chest X-ray or ULD chest CT, will be performed (Fig. 1). At AMC, ULD chest CT will be performed at a Siemens Somatom Force (Siemens Healthineers, Erlangen, Germany) with fixed 100 kV, Sn filter, and reference 50 mAs; in SG at a Toshiba Aquilion One (Toshiba Medical Systems Corporation, Otawara, Japan) with fixed 120 kV and 10 and 20 mAs in adipose patients. Chest X-ray is performed following standard procedures. At AMC, chest X-ray will be performed on an Oldelft bucky (Oldelft Benelux B.V., Veenendaal, the Netherlands) at 125 kV (0.2 mm Cu filter) using automated exposure control and at SG Siemens Ysio Max at 125 kV (0.2 mm CU filter) using automated exposure control. Chest X-ray will be performed on a posterior/anterior and lateral direction whenever possible. If not possible, anterior/posterior chest X-ray will be performed using a mobile chest X-ray device Carestream DRX Revolution (Carestream Inc., Rochester, NY) in all participating centers.

All patients that can undergo a chest X-ray posterior anterior (PA), or anterior posterior (AP) if clinically indicated, and CT are included in the study. If possible, patients will get a PA and lateral chest X-ray. Because of the known difference in the image quality between PA and AP chest X-ray, we will analyze the differences in the outcomes between the PA and AP patient groups.

Structured standardized reporting of the images will be performed, and the examinations will be read or supervised by radiologists experienced in chest radiology, also outside regular office hours. All relevant findings will be documented, including incidental findings, cardiac calcifications, and skeletal findings.

The radiologist will formulate a radiologic diagnosis and probability. The results of the chest X-ray or ULD chest CT will be communicated directly with the attending physician, after which the attending physician will formulate a final clinical diagnosis (Fig. 1).

If the clinical question is not adequately answered after obtaining the chest X-ray or ULD chest CT, standard additional imaging (e.g., chest CT with intravenous contrast medium, CT pulmonary angiography) will be performed. If there is a high suspicion of pulmonary emboli, patients will only get a CT pulmonary angiography, in accordance with regular clinical practice. Initial and subsequent treatment of CAP, including antibiotic treatment, duration of treatment, and discharge from the hospital, will be at the discretion of the attending physician, according to current Dutch guidelines [16, 27].

In patients suspected of CAP, additional laboratory tests are done: blood and sputum cultures, urine pneumococcal and *Legionella* antigen test (the latter if CURB-65 score is > 2), and naso- and pharyngeal swab for respiratory pathogens by polymerase chain reaction (PCR).

Clinical history, physical examination, clinical diagnosis at discharge from the ED, time to preliminary imaging result, time spent at ED, events possibly related to the study procedure, and results of laboratory and radiological examinations will be coded and saved in the electronic case record forms (eCRF) (Castor EDC, Amsterdam, the Netherlands) by research nurses and medical students (Table 1).

**Table 1:** SPIRIT OPTIMACT study scheme

Time scheme OPTIMACT study			
	Enrolment	Allocation	
Time point	*t*_0_	*t*_0_	*T*_28_
Enrollment	Day 0 ED	Day 0 ED	Day 28
Eligibility screen	X		
Informed consent	X		
Allocation		Block randomization per month	
Interventions			
Intervention A: chest X-ray		A or B	
Intervention B: ULD chest CT			
Assessments			
Standardized clinical history		X	
Standardized physical examination		X	
Predefined laboratory set: WBC, glucose, urea, and CRP		X	
Suspected of CAP Laboratory set etiology pneumonia		X^*^	
Standardized radiology report		X	
Clinical diagnosis at discharge from ED		X	
Suspected of CAP Treatment decision regarding antibiotic use*		X^*^	
Events possibly related to the study procedure		X	
Time to preliminary imaging result		X	
Time spent at ED		X	
Diagnosis at discharge based on data day 28			X
Length of hospital stay			X
Suspected of CAP Antibiotic use			X^*^
Follow-up because of incidental findings on chest X-ray or ULD chest CT			X
Mortality 28 days			X
SF12/EQ-5D-5L			X
Health care costs			X
iMCQ			X
iPCQ			X

^*^Additional assessments for patients suspected of CAP

### Follow-up

#### Clinical follow-up

Data will be obtained from the electronic patient record after 28 days, including course of the disease, treatment outcome, length of hospital stay, mortality, laboratory findings, antibiotic use, and follow-up because of incidental findings on chest X-ray or ULD chest CT (Table 1). In patients referred to another hospital, data will be collected at that hospital.

#### Questionnaires follow-up

After 28 days, patients are called to remind them of the questionnaires that will be sent via mail or email depending on the patient’s preference. Functional health is assessed using the SF-12/EQ-5D-5L. The health care costs associated with medical consumption and productivity loss due to sick leave from work are assessed by the Institute for Medical Technology Assessment (iMTA), Medical Consumption Questionnaire (iMCQ) and Productivity Cost Questionnaire (iPCQ), both adapted to the study setting (Table 1). In the iMCQ, patients are asked whether they have consulted a general practitioner (GP) or visited a hospital in relation to their visit to the ED. If so, clinical follow-up information is collected at the GP or hospital.

### Criteria for clinical diagnoses at day 0 and day 28

ED discharge diagnoses (day 0 diagnosis) will be derived from the conclusions by the attending physician at discharge from the ED.

The final post hoc diagnoses will consider all available clinical, radiological, and microbiological data at 28 days of follow-up. A diagnostic handbook has been developed for the OPTIMACT study to define these post hoc diagnoses, enabling standardized and reproducible categorization. The handbook describes diagnostic reference standards for frequently occurring non-traumatic thoracic diseases like influenza, pneumonia, cardiac failure, etc. Each patient will be reviewed independently by two reviewers using the diagnostic handbook. In case of disagreement, a third reviewer (a physician with at least 1 year of clinical experience) will independently evaluate the patient as well. If the three reviewers still disagree after the case has been discussed in a plenary discussion, or if the diagnostic handbook indicates referring the case because of complexity, a final diagnosis will be made by an independent adjudication committee consisting of specialists in internal medicine, pulmonology, cardiology, and chest radiology. The members of the adjudication committee should not have been involved in the care of the study patients of AMC or SG, respectively, during the study period. The use of this diagnostic handbook has been tested in two pilot studies, and interobserver agreement will be evaluated in a final validation study. This methodology is adapted from previously published work and will be reported in a separate paper [32–34].

### Outcome measures

#### Primary outcome

Functional health at 28 days is the primary clinical outcome. Functional health at 28 days is measured by the physical summary scale of the SF-12. This simple, generic health-related quality of life instrument is selected because of the diversity of diseases to be encountered.

#### Secondary outcome

Secondary outcomes are mental health (measured by the mental summary scale of the SF-12), length of hospital stay, mortality within 28 days, quality-adjusted life year (QALY) during the first 28 days (based on EQ-5D-5L health status scoring profiles), correct diagnoses at ED discharge as compared to the final post hoc diagnosis at day 28, number of patients in follow-up because of incidental findings on chest X-ray or ULD chest CT, and health care costs.

For patients suspected of CAP, additional secondary outcomes are correct diagnosis of CAP at ED discharge as compared to the final diagnosis at day 28, initial treatment decision of the attending physician regarding antibiotic treatment, total antibiotic use over 28 days, etiology of pneumonia in patients with CAP, and correlation of etiology with results of the chest X-ray and ULD chest CT.

### Data analysis

We will test the null hypothesis of non-inferiority of ULD chest CT against chest X-ray in terms of the absence of a meaningful difference (0.1 margin in the mean score) in functional health at 28 days. We will use a one-sided hypothesis test based on the two-sample *t* test statistic. Given the pragmatic nature of the trial, the main analysis will be done for the intention-to-imaging (ITI) population. Considering the non-inferiority study design, this analysis will be complemented by an auxiliary analysis performed in the imaging-per-protocol (IPP) population. The ITI population includes all patients referred for imaging and fulfilling the inclusion and exclusion criteria set. The IPP population includes all patients that actually received the initial imaging procedure according to the randomization scheme, thereby excluding patients who were offered another diagnostic trajectory for hospital logistic reasons (e.g., multiple patients presenting simultaneously). For the analysis of the health care costs, see the “Economic evaluation” section below.

Secondary outcomes will be assessed in the ITI population by parametric and non-parametric descriptive analyses, depending on data distributions. Diagnostic test characteristics of chest X-ray and ULD chest CT will be reported according to the STARD guidelines [35].

An exploratory regression analysis, with baseline characteristics of patients, will be performed to identify variability in benefit (or harm) from replacing chest X-ray by ULD chest CT. This may identify subgroups of patients who clearly benefit, or who lack a benefit, from replacing chest X-ray by ULD chest CT.

### Sample size

The hypothesis is that introducing ULD chest CT will reduce the costs while being at least equivalent to chest X-ray regarding functional health. A standard deviation of 10 is anticipated [36]. To have 80% power to exclude a 0.1 difference in the mean SF-12 score, two-sample *t* test statistic, 2400 participants are needed; this small non-inferiority margin comes down to a 0.01 effect size.

Given an anticipated 63% inclusion rate (based on pilot data), 3810 potentially eligible patients are needed. Every month, 705 potentially eligible patients are seen in both hospitals combined. As the study started earlier at the AMC, we will not aim at an equal contribution of both hospitals. When 2400 inclusions are reached, the calendar month will be completed after which enrolment will stop.

### Economic evaluation

The economic evaluation of ULD chest CT versus chest X-ray in patients suspected of non-traumatic pulmonary disease will be performed from a societal perspective as a cost-utility analysis. The time horizon is restricted to a follow-up of 28 days because treatment patterns beyond this horizon will very likely be independent of the choice of the initial imaging procedure and tend to neutralize differences in health outcomes.

An incremental cost-utility ratio (ICUR) will be calculated reflecting the extra costs per additional QALY (see below) for ULD chest CT versus chest X-ray. It will be graphically presented along with its 95% confidence ellipse, following bootstrapping. A cost-effectiveness acceptability curve will show the probability at various levels of the societal willingness to pay per QALY up to 80,000 euros. In the likely case of near equivalence of both imaging strategies in QALYs, rendering the ICUR to infinity, a cost-minimization analysis will additionally be performed. Differences in health care costs will be presented descriptively along with their 95% confidence intervals, again following bootstrapping. A positive outcome of the exploratory regression analysis mentioned will allow further analysis of homogeneous subgroups.

All relevant medical costs, patient/family costs, and costs of productivity loss due to sick leave from work will be calculated with resource use data gathered from clinical report forms, available hospital information systems (EPIC), and the iMCQ and iPCQ adjusted to the study setting (to be completed by patients at day 28) and with unit costing complying with the Dutch guideline on costing in health care research [37] .

Patients’ functional health will be assessed with the SF-12 at day 28, the primary clinical outcome measure. In addition, the EQ-5D-5L questionnaire will also be applied at day 28 to assess a patient’s health status. The EQ-5D-5L health status profiles will subsequently be transposed in quality-adjusted life years by applying existing Dutch time trade-off-based health utility algorithms divided by 13.04 (365.25/28) [38].

The economic evaluation will be reported according to the CHEERS reporting guideline [39].

Results of the trial will be published in peer-reviewed journals and presented at scientific meetings.

## Discussion

Our study evaluates the impact of replacing chest X-ray by ULD chest CT on a population basis. The diagnostic superiority of chest CT compared to chest X-ray is already well established [2–9]. The image quality and radiation dose of ULD chest CT are less than the standard chest CT, but ULD chest CT gives a high level of diagnostic confidence for patients suspected of pulmonary disease at the ED [22]. Our aim is to answer the question whether more accurate imaging will result in increased efficiency and lower costs, with at least comparable health-related quality of life.

A multicenter, pragmatic, block-randomized controlled trial has been chosen to make the study feasible in a 24/7 clinical setting. Randomization per patient would probably prohibit the inclusion of the most seriously ill patients, thereby introducing selection bias.

Diagnostics, both clinical and radiological, will be performed by the residents and specialists on call. This reflects daily practice in the participating hospitals and will make our results more generalizable. Structured reporting of the chest X-ray and ULD chest CT will contribute to a uniform evaluation and reporting of the images.

By the participation of a university and a large teaching hospital, a broad spectrum of patients will be included. In addition, the AMC and SG have ethnically diverse populations. The broad definition of patients suspected of non-traumatic pulmonary disease results in the inclusion of cases across the whole clinical spectrum of patients with acute pulmonary complaints, yielding results applicable to the general population presenting with non-traumatic pulmonary disease at the ED.

In our study, we also evaluate whether the improved diagnostic accuracy of ULD chest CT will result in a better prediction of the etiology of the CAP compared to chest X-ray and whether improved diagnostics results in better management. Incidental findings may lead to additional examinations, which increase costs and uncertainty, without contributing to the patient outcome in most patients. The follow-up period of 28 days is too short to evaluate the final impact of incidental findings but was chosen based on the previously described primary outcome measures. Especially, pulmonary nodules are a major source of concern. One study on chest CT findings performed between 2006 and 2012 in the general population reported 24 to 31% nodules in all scans [28]. Depending on the aspects of the pulmonary nodule, the follow-up will take 3 months to 5 years [40]. The study follow-up period is 28 days; therefore, because of the incidental findings in the chest X-ray and ULD chest CT groups, only the number of patients in follow-up will be reported.

This pragmatic study will provide evidence on the effects of replacing chest X-ray by ULD chest CT in daily practice, in terms of patient-related health outcomes and costs, in the diagnostic work-up of patients suspected of non-traumatic pulmonary disease at the ED.

## Additional files

Additional file 1: Appendix 1. SPIRIT OPTIMACT checklist. (PDF 56 kb)

Additional file 2: Appendix 2. Patient information folder regular version. (PDF 61 kb)

Additional file 3: Appendix 3. Patient information folder short version. (PDF 42 kb)

## Abbreviations

AMC: Academic Medical Center
Amsterdam UMC: Amsterdam University Medical Centers
CAP: Community-acquired pneumonia
CEA: Cost-effectiveness analysis
CT: Computed tomography
eCRF: Electronic case record forms
ED: Emergency department
ICUR: incremental cost-utility ratio
iMCQ: iMTA Medical Consumption Questionnaire
iMTA: Institute for Medical Technology Assessment
iPCQ: iMTA Productivity Cost Questionnaire
ITI analysis: Intention-to-imaging analysis
MEC: Medical Ethics Committee
QALY: Quality-adjusted life year
SG: Spaarne Gasthuis
ULD chest CT: Ultra-low-dose chest computed tomography
