# Supplementary material for: OPTimal IMAging strategy in patients suspected of non-traumatic pulmonary disease at the emergency department: chest X-ray or ultra-low-dose CT (OPTIMACT)—a randomised controlled trial chest X-ray or ultra-low-dose CT at the ED: design and rationale
Source: Diagn Progn Res. 2018 Aug 8;2:20. doi: 10.1186/s41512-018-0038-1 (PMC6460797; doi:10.1186/s41512-018-0038-1)
Supplement: Supplementary file 2 — Appendix 2. Patient information folder regular version. (PDF 61 kb) [file 41512_2018_38_MOESM2_ESM.pdf]

Subject information of:

Optimal imaging strategy in patients suspected of pulmonary disease: Chest X-ray or CT
The OPTIMACT trial

Official title:
Optimal imaging strategy in patients suspected of non-traumatic pulmonary disease at the Emergency Department: Chest X-ray of CT
The OPTIMACT trial

Dear Sir/Madam,

You have received this letter because you are visiting the Emergency Department of the AMC because of pulmonary complaints. The attending physician of the Emergency Department will order a radiologic chest examination to have more insight into the cause of your complaints.

At this moment there are two radiologic chest examinations:
1) Chest X-ray
2) Chest CT
Chest X-ray has a low radiation dose and is generally available. Chest CT provides substantially more detailed information on conditions that cause pulmonary complaints, but until recently the radiation dose was much higher. Because of that high radiation dose often a chest X-ray was ordered first. Recent advances in CT-technology have resulted in a chest CT (ultra-low-dose chest CT) with a radiation dose, comparable to two chest X-rays. The radiation dose of this CT is not an issue anymore and therefore it is possible to directly start with an ultra-low-dose chest CT. Both imaging techniques are generally accepted imaging techniques but not studied yet as we do, namely in the setting of the Emergency Department. We want to investigate whether it is preferable to skip chest X-ray and directly perform an ultra-low-dose chest CT in patients with complaints of the lungs .

If you participate in this study, depending on the month in which you visit the Emergency Department of the AMC a chest X-ray or ultra-low-dose chest CT will be made. We would like to use your medical information for research purposes to investigate the radiologic imaging technique of choice for patients with pulmonary complaints at the Emergency Department.

The study is explained in this form. Please read this information carefully and ask the investigators for an explanation if you have any questions. You may also discuss it with your partner, friends or family. If you agree that your medical information is used for this research purpose, please fill in the subject consent form (appendix 1).

What is the purpose of the study?

The purpose of this study is to investigate for which patient population ultra-low-dose chest CT had added value. It seems a logic step to replace ultra-low-dose chest CT for chest X-ray because with ultra-low-dose chest CT the lungs can be evaluated in more detail. This will most likely lead to more timely diagnoses and improved patient management. There are some limitations in the use of ultra-low-dose chest CT, its availability is limited and the costs are higher. More lung nodules will be detected and patients will need additional investigations to identify the nature of the lung nodules. Only a minority of patients will benefit from these investigations. Therefore it is necessary to compare both strategies to determine whether it is effective to replace chest X-ray for ultra-low-dose CT in patients suspected of lung disease at the Emergency Department.

How is the study performed?

Each month a computer randomly selects the first imaging method of choice (chest X-ray or ultra-low-dose chest CT), for patients with complaints of the lungs at the Emergency Department who will participate in this trial.

During your visit of the Emergency Department your medical condition is assessed. Your physician does this by the evaluation of your medical complaints, information given by the nurse, physical examination and

laboratory tests. Based upon this information imaging of the chest is indicated. The attending physician or research nurse ask your permission for participation in the OPTIMACT trial, depending on the month of presentation in the AMC a chest X-ray or ultra-low-dose chest CT is performed. Both imaging methods are comparable in duration (5 minutes) and burden. Both investigations are part of routine care for patients with pulmonary complaints and both are accepted imaging methods.

We ask your permission for using your medical information of your stay at the Emergency Department and the first 28 days thereafter for this study. You will receive a questionnaire (with a maximum of 15 minutes) 28 days after your visit to the Emergency Department. The questionnaire can be send by regular post or by email. If you are willing to receive the questionnaire by email you can fill in your email address on the subject consent form. A research nurse will contact you by phone or email around that time to ask a few additional short questions.

What does participation mean for you?
To be able to improve patient care for patients with pulmonary diseases it is very important that an adequate number of patients are willing to participate in scientific research involving pulmonary diseases. More knowledge results in improvement of diagnostic strategies of pulmonary conditions and subsequently improving treatment results.
There are no advantages or disadvantages when participating. You will undergo the regular treatment and care, this will not change if you decide not to participate in this study.
Participation is not associated with additional costs. There is no compensation for participation in the OPTIMACT trial. You will not be paid for your participation in this study.

Which side effects can you expect?
There are no additional investigations in this trial and therefore no side effects can be expected. Chest X-ray and ultra-low-dose chest CT are accepted imaging methods. Both imaging techniques are radiologic techniques associated with an average radiation dose of 0.05 mSv for chest X-ray and

0.1 mSv for ultra-low-dose chest CT. If we compare this dose to the annual background radiation in the Netherlands this is 2-2.5 mSv.

If you do not want to participate or you want to stop participating?
Participation is completely voluntary. If you do not want to participate, you will be treated as usual for your disease. If you do participate in the study, you can always change your mind. You may stop participation at any time during the study. It will not influence your medical treatment. You do not have to say why you withdraw.

Are you insured when you participate?
As both Chest X-ray and ultra-low-dose chest CT are accepted diagnostic methods participation is not associated with risks., The Institutional Review Board of the AMC has waived the need for a study participants insurance.

What is done with your data and bodily material?
For this study it is necessary to collect and use your medical and personal data. All personal identifying information (e.g. name, address) will be deleted and a code will be used. This code will be used for labelling the data.

Your data
All your data will remain confidential. The investigators and research assistants are the only people who will know the code you have. The key to the code will stay with the investigators. Also in the reports about the study only this code will be used.

Some other people may access your data. This is to check whether the study is done adequate and reliable. These people are representatives of the AMC or the National Inspection of Health Care (‘Inspectie voor de Gezondheidszorg’).General information about this can be found in the general brochure on medical research (appendix 2).
The study results will be published. In these publications your personal information will not be identifiable.
It is mandatory that the investigator stores your data for 15 years. If you sign the consent form, you consent to your medical and personal data being

collected, stored and accessed.

Which Institutional Review Board has consented for this trial?

The Institutional Review Board of the Academic Medical Centre Amsterdam has consented for this study. This study is listed in the Dutch trial registry under number NTR6163. This website does not contain any information that can identify you.

General information about the registration of research can be found in the general brochure on medical research (appendix 2).

Do you want to know more?

If you have any questions, please contact the investigator, research assistent or attending physician. (You find names and contact information at the bottom of this letter.) Also during the study when you have questions or encounter a problem it is possible to ask questions to the investigator, research assistent or your own physician.

In addition, if you do not want to ask questions to the investigators before and during the study you can contact an independent physician, prof. M. Maas, radiologist. The independent physician is not involved in this study but he is knowledgeable concerning this study and your disease. If you have doubt about participation you can also ask the independent physician for advice.

Signing the subject consent form

If you decide to participate we will ask you to sign the subject consent form (appendix 1). By signing the consent form you confirm your intention to participate in this trial. You are free to withdraw at any time. The attending physician, investigator or research assistant will also sign the form confirming that he or she has informed you about the trial, has given you the information brochure and is willing to answer all your questions.

How to act in case of complains?

If you have any complaints, you can report this to the investigator or your attending physician, you may also contact the patient information desk of the AMC.

Contact details

Investigator:
Prof. dr. J. Stoker, radiologist, phone: 020-5662532
Drs. I.A.H. van den Berk, radiologist, phone: 020-5664093
Research assistents:
Drs. E.M. Taal, phone: 020-5663229
Mw. D. Hulzebosch, phone: 020-5663229
Mw. A. Schoonderwoerd, phone: 020-5663229

Independent doctor: Prof. M. Maas, radiologist, phone: 020-5663629
Patient information desk AMC: 020-5663355

Thank you for your attention.

Appendices

1. Subject consent form
2. General brochure on medical research

Subject Consent Form

Optimal imaging strategy in patients suspected of pulmonary disease:

Chest X-ray or CT

The OPTIMACT trial

I have read the subject information form. I was also able to ask questions. My questions have been answered to my satisfaction. I had enough time to decide whether to participate.

I know that participation is voluntary. I know that I may decide at any time not to participate after all or to withdraw from the study. I do not need to give a reason for this.

I know that some people can access my data. These people are listed in this information.

I give permission for using my medical and personal data for the purposes mentioned in the subject information letter.

I consent to my data being stored for 15 years after this study.

I want to participate in this study.

Name of study subject:

Signature: Date: __ / __ / __

I want to receive the questionnaire by email.

Email address:…………………………………@..

I hereby declare that I have fully informed this study subject about this study.

If information comes to light during the course of the study that could affect the study subject's consent, I will inform him/her of this in a timely fashion.

Name of investigator/physician:

Signature: Date:__ / __ / __

Time:
