# Supplementary material for: OPTimal IMAging strategy in patients suspected of non-traumatic pulmonary disease at the emergency department: chest X-ray or ultra-low-dose CT (OPTIMACT)—a randomised controlled trial chest X-ray or ultra-low-dose CT at the ED: design and rationale
Source: Diagn Progn Res. 2018 Aug 8;2:20. doi: 10.1186/s41512-018-0038-1 (PMC6460797; doi:10.1186/s41512-018-0038-1)
Supplement: Supplementary file 3 — Appendix 3. Patient information folder short version. (PDF 42 kb) [file 41512_2018_38_MOESM3_ESM.pdf]

Optimal imaging strategy in patients suspected of non-traumatic Pulmonary disease Chest X-ray or CT
The OPTIMACT trial

If you participate in this study, depending on the month in which you visit the Emergency Department of the AMC a chest X-ray or ultra-low-dose chest CT will be performed.

Chest X-ray has a low radiation dose and is generally available.
Recent advances in CT-technology have resulted in a chest CT (ultra-low-dose chest CT) with a low radiation dose.
We want to investigate whether it is preferable to skip chest X-ray and directly perform an ultra-low-dose chest CT in patients with complaints of the lungs.
Both imaging methods are comparable in duration (5 min) and burden.

Patient consent for:

A Chest X-ray (X-thorax)
or
a Chest CT (CT-thorax)

O I do agree that at random one of the above mentioned examinations is performed

…………………………………………………….
Signature witness
Name and function:

After leaving the Emergency Department you will receive more information.

Name of investigator
Signature: Date:__ / __ / __
Time:……….
